# Accelerating the development of genome-edited crops and the establishment of utilization infrastructure

**DOI:** 10.1270/jsbbs.74.1

**Published:** 2024-03

**Authors:** Hiroshi Ezura

**Affiliations:** 1 Faculty of Life and Environmental Sciences, University of Tsukuba

Genome-editing technology, a biological mutation induction technique, is attracting attention as a novel and effective tool for mutation breeding. In particular, after the report on CRISPR/Cas9 technology (Cong *et al.* 2013), a simple and efficient genome-editing technology, expectations for this technology have increased further. In Japan, the Cross-ministerial Strategic Innovation Promotion Program (SIP: 2014–2018), a national project to verify the effectiveness of genome-editing technology in improving agricultural, forestry, and fishery products, was carried out, and genome-edited crops (tomato, potato, rice, wheat, etc.) and fish (tuna, red sea bream, puffer fish, etc.) that are expected to be put to practical use have been developed.

From these, regulatory filings for genome-edited tomatoes were completed at the end of 2020, and they began to be marketed to consumers in September 2021 and in supermarkets in 2023, becoming the first agricultural products developed using CRISP/Ca9 technology to be marketed in supermarkets in the world (Ezura 2022, Waltz 2022). Such developments and social implementation of genome-edited crops in Japan are leading the way for the social implementation of genome-edited crops around the world. Furthermore, social acceptance of genome-edited crops is also changing. Even in Europe, the most conservative region in terms of the regulation of genome-edited crops, new regulation rules for genome-edited crops were proposed in July 2023, and deliberations are underway. In conjunction with these developments, the development of new genome-editing technologies and genome-edited crops is gaining momentum around the world.

This special issue of *Breeding Science* introduces global trends in the regulation rules for genome-edited crops (foods) (Tachikawa and Matsuo 2024), trends in the social license for genome-edited crops in Japan (Yamaguchi *et al.* 2024), the development of new genome-editing technologies (Hozumi *et al.* 2024, Ikeda 2024), and genome-edited crops that are expected to be implemented in society in the future: wheat (Kaur *et al.* 2024), melon (Nonaka and Ezura 2024), and tomato (Nagamine and Ezura 2024). The development of genome-editing technology and genome-edited crops will be introduced, and I hope to provide some new ideas for researchers working on the development of these.

I thank the authors of the articles featured in this special issue for their contributions and thoughtful insights into current advances in this research field. These articles provide valuable information that can be used in future plant breeding. Finally, I would like to thank the Editor-in-Chief, Dr. Tsujimoto, for giving me the opportunity to edit this special issue.
